# High body mass index, brain metabolism and connectivity: an unfavorable effect in elderly females

**DOI:** 10.18632/aging.102347

**Published:** 2019-10-09

**Authors:** Arianna Sala, Maura Malpetti, Anna Ferrulli, Luigi Gianolli, Livio Luzi, Daniela Perani, Alzheimer’s Disease Neuroimaging Initiative (ADNI) database

**Affiliations:** 1 Vita-Salute San Raffaele University, Milan, Italy; 2 In vivo Human Molecular and Structural Neuroimaging Unit, Division of Neuroscience, IRCCS San Raffaele Scientific Institute, Milan, Italy; 3 Metabolism Research Center and Endocrinology and Metabolism Division, IRCCS Policlinico San Donato, Milan, Italy; 4 Nuclear Medicine Unit, IRCCS San Raffaele Hospital, Milan, Italy; 5 Università degli Studi di Milano, Milan, Italy

**Keywords:** body mass index, connectivity, PET, brain, gender

## Abstract

There are reported gender differences in brain connectivity associated with obesity. In the elderlies, the neural endophenotypes of obesity are yet to be elucidated. We aim at exploring the brain metabolic and connectivity correlates to different BMI levels in elderly individuals, taking into account gender as variable of interest.

We evaluated the association between BMI, brain metabolism and connectivity, in elderly females and males, by retrospectively collecting a large cohort of healthy elderly subjects (N=222; age=74.03±5.88 [61.2-85.9] years; M/F=115/107; BMI=27.00±4.02 [19.21-38.79] kg/m^2^). Subjects underwent positron emission tomography with [18F]FDG. We found that, in females, high BMI was associated with increased brain metabolism in the orbitofrontal cortex (R=0.44; p<0.001). A significant BMI-by-gender interaction was present (F=7.024, p=0.009). We also revealed an altered connectivity seeding from these orbitofrontal regions, namely expressing as a decreased connectivity in crucial control/decision making circuits, and as an abnormally elevated connectivity in reward circuits, only in females. Our findings support a link between high BMI and altered brain metabolism and neural connectivity, only in elderly females. These findings indicate a strong gender effect of high BMI and obesity that brings to considerations for medical practice and health policy.

## INTRODUCTION

Obesity differs in women and men for several aspects. First, the prevalence of obesity is higher in women (38.3%) than in men (34.3%) and gender difference in prevalence of obesity is constant across different age and race groups [1]. Second, the phenotype of female obesity is different from that of male obesity [2]. The gender difference in body composition might be due to hormonal, environmental, psychological and/or dietary factors [2]. Finally, consistent evidence supports the theory that dietary patterns are different among sexes, as shown by Forster et al. [3]. According to Westenhoefer [4], food choices and eating behavior show gender differences. It was also shown that males and females crave for different foods [5]. Response to sweet taste was reported stronger in men than women, with different degrees of activation of the caudate nucleus across sexes, as shown by functional magnetic resonance imaging (fMRI) [6].

The explanation for gender differences in dietary habits is still under debate. The most likely reasons imply either the effects of sex hormones on brain response to food [6] or an intrinsic gender difference in astrocytes physiology [7] or in brain wiring, as shown by a study on white matter morphology [8]. Different dietary habits between sexes may determine the different prevalence rates in the overall population of women and men with obesity.

Former fMRI neuroimaging studies reported sex/gender differences in neural correlates of food stimuli. Chao et al. reported higher activation in women with obesity than in men with obesity in frontal, limbic, striatal areas and fusiform gyrus brain regions in response to visual food cues [9]. These consistent findings suggest a female-specific impairment in the inhibitory control systems in obesity. Exposure to appetitive food stimuli is known to activate human brain as shown also by [18F] Fluoro Deoxy Glucose (FDG) PET [10]. Using the same methodology, Wang and colleagues were able to demonstrate a gender difference in cognitive inhibition capacity [11], with males more effective than females in voluntary constraining food intake.

Only few studies investigated obesity-related changes in brain metabolism in healthy adults [12]. For example, Volkow et al. [13] reported a negative correlation between Body Mass Index (BMI) levels and brain metabolism of prefrontal cortex and cingulate gyrus in a group of young and healthy subjects. Wang et al. [14] reported higher metabolic activity in obese than lean subjects in postcentral gyrus of the parietal cortex. However, to the best of our knowledge, the relation between BMI levels and brain metabolic changes in heathy elderlies has not been explored yet.

Based on the quoted literature evidence, we aimed to explore the brain metabolic correlates to different BMI levels in elderly individuals. Especially, we investigated the gender differences in the association between BMI and FDG-PET measures in a group of 222 elderly subjects, as part of the Alzheimer’s Disease Neuroimaging Initiative (ADNI). We hypothesized a significant relationship between BMI levels and brain metabolism, and connectivity in the elderly population, with gender-specific differences.

## RESULTS

### Gender-specific association between brain metabolism and BMI

### Voxel-based analysis

**Figure 1:**
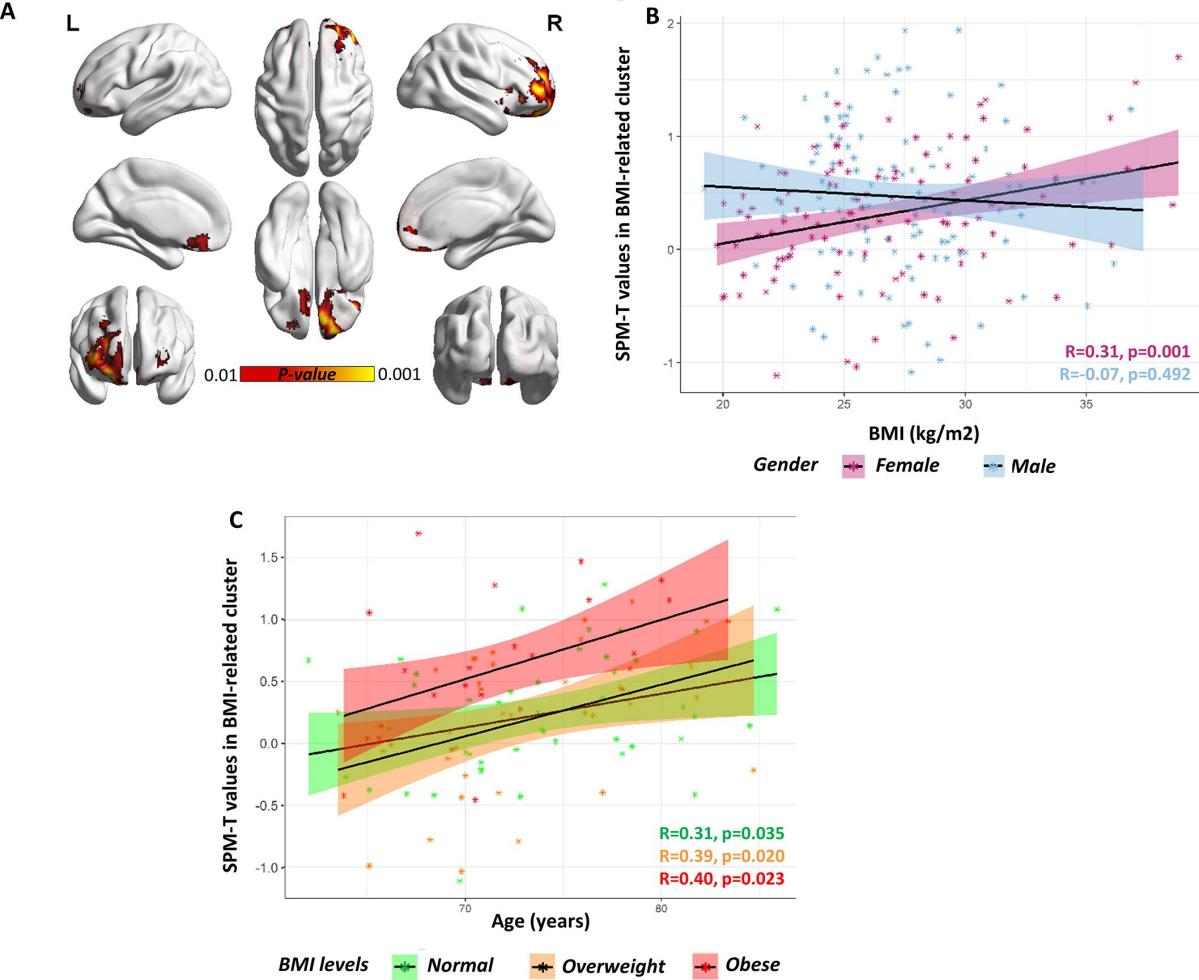
**Gender-specific voxel-wise correlation between BMI levels and brain metabolism.** (**A**) A significant positive correlation was found in orbitofrontal regions (partial R=0.44) in females. Statistical threshold was set at p<0.001 (uncorrected for multiple comparisons), with minimum cluster extent Ke:100 voxels (yellow). For visualization purposes, figure also shows voxels where correlation is significant at a more liberal threshold (p<0.01 uncorrected for multiple comparison; red). For both p<0.001 and p<0.01 voxel-level thresholds, only clusters surviving p<0.05 FWE-correction are shown. BrainNet Viewer (http://www.nitrc.org/projects/bnv/) was used for rendering [52]. (**B**) Scatter plot shows the significant BMI by gender interaction on orbitofrontal metabolism, with females showing a significant positive correlation between BMI levels (x axis) and average SPM-T values of glucose metabolism (y axis) (R=0.31, p<0.001; partial R=0.44, p<0.001) and males showing no correlation at all (R=-0.07, p=0.492; partial R=-0.01, p=0.881). Positive SPM-T values indicate higher-than-average mean orbitofrontal glucose metabolism: in females, higher BMI levels are associated with increased orbitofrontal glucose metabolism, crucially approaching critical hypermetabolism levels in the case with highest BMI levels (BMI ≈ 40kg/m2). Shaded areas represent confidence intervals for the regression line slope in each group. (**C**) Scatter plot shows the lack of a significant BMI by age interaction on orbitofrontal metabolism, despite of a significant principal effect of age (partial R=0.32, p<0.001), in the female cohort. The slope of the regression lines in the different (normal, overweight and obese) BMI groups does not differ: there is no significant interaction effect between age and BMI, but both age and BMI have an independent effect on orbitofrontal metabolism. Age (years) is plotted on the x axis and metabolism on the y axis. Shaded areas represent confidence intervals for the regression line slope in each group.

ANCOVA showed no significant association between brain metabolism and BMI in the male group. In the female group instead, we found that BMI significantly predicted brain metabolism, with higher BMI associated with increased brain metabolism in orbitofrontal regions, peaking in the right superior orbitofrontal gyrus at MNI coordinates (x, y, z) 8 44 -28, and remaining significant at p<0.05 FWE-corrected at the cluster-level. This cluster encompassed the right lateral orbitofrontal cortex, and extended dorsally to the rostro-polar portions of the right middle and superior frontal gyri (BA 9; 10; 46) (see Figure 1A, Table 1). Thus, in this cluster, high BMI levels were associated with increased metabolism (partial R=0.44; p<0.001), also surpassing the critical threshold for hypermetabolism (T>1.65) in a subject with morbid obesity (BMI = 38.79 kg/m^2^) (Figure 1B).

**Table 1:** Clusters where glucose metabolism is significantly predicted by BMI levels (female cohort).

**Cluster- level**			**Voxel-level**					
**P-value (FWE-corr.)**	**Extent**	**P-value (unc.)**	**P-value (FWE-corr.)**	**P-value (FDR-corr.)**	**T score**	**Z score**	**P-value (unc.)**	**MNI coordinates x, y, z {mm}**
**p<0.001 (cluster-level threshold: 0.05 FWE-corrected, Ke: 100 voxels)**								
<0.001	1029	<0.001	0.087	0.075	4.59	4.48	<0.001	8 44 -28
			0.358	0.075	4.14	4.06	<0.001	22 58 -14
			0.556	0.080	3.96	3.89	<0.001	14 54 0
**p<0.01 (cluster-level threshold: 0.05 FWE-corrected, Ke: 100 voxels)**								
<0.001	6743	<0.001	0.077	0.075	4.62	4.51	<0.001	-34 60 8
			0.087	0.075	4.59	4.48	<0.001	8 44 -28
			0.324	0.075	4.18	4.09	<0.001	-10 36 -12

Results are shown at p<0.001 and p<0.01, uncorrected for multiple comparisons (cluster-level threshold: 0.05 FWE-corrected, Ke: 100 voxels). Three local maxima more than 8.0mm apart are shown for each cluster.

Post-hoc analysis revealed a significant BMI x gender interaction on average cluster metabolism (F=7.024, p=0.009), suggesting that the effect of BMI on cluster metabolism is significantly stronger in females as compared to males (Figure 1B). No significant BMI x age interaction was found (F=2.342, p=0.127), even though both BMI and age were significant predictors of metabolism in the orbitofrontal BMI-related cluster (F= 13.52, p<0.001; F=6.446, p=0.012, respectively) in the female cohort (Figure 1C). This suggests that age and BMI are both significant and *independent* predictors of metabolic function in orbitofrontal regions (only in females), and that their effects on metabolism combine in an additive fashion.

### Region of Interest (ROI)-based analysis

**Figure 2:**
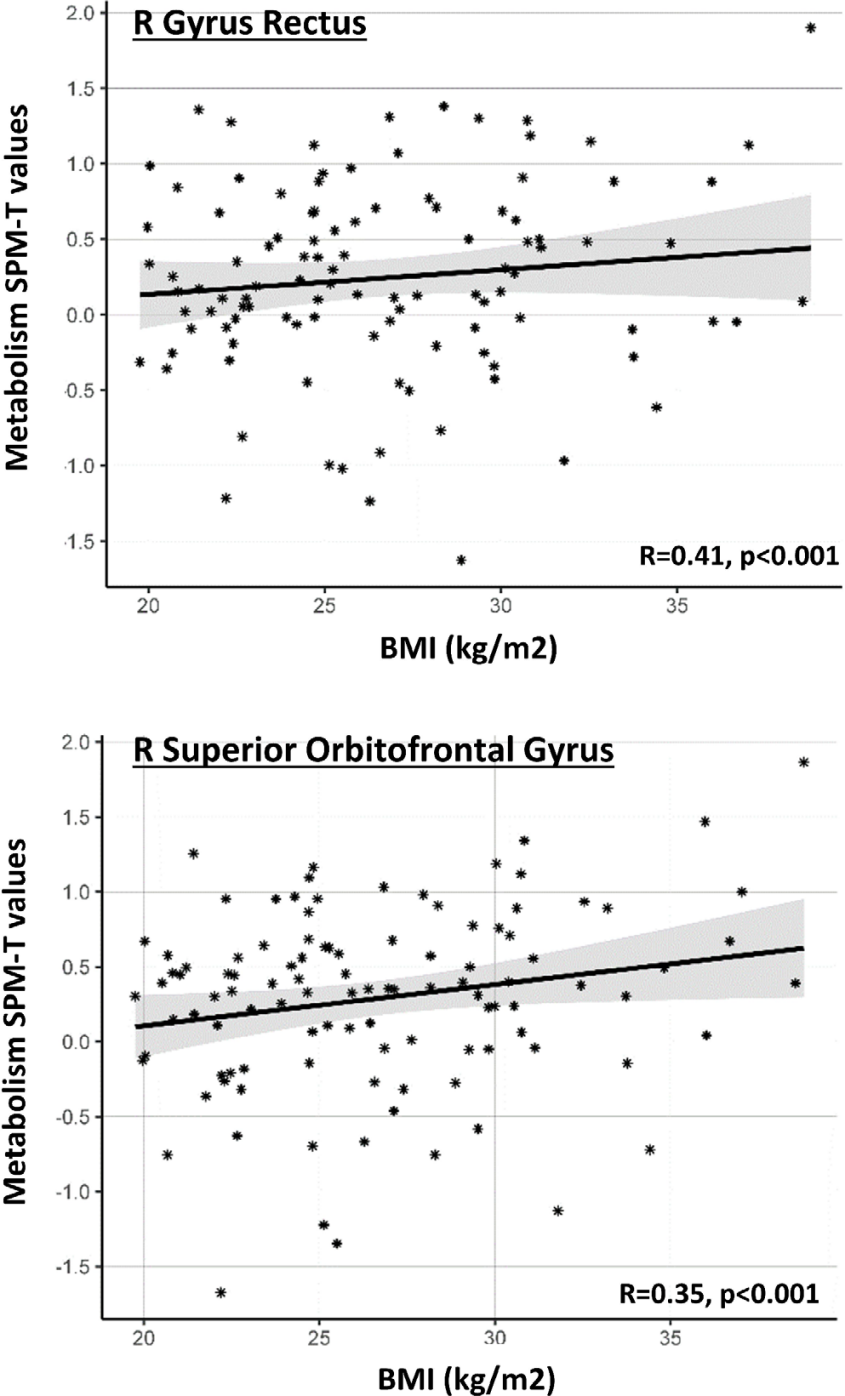
**Gender-specific ROI-based correlations between BMI levels and regional metabolism.** Graph shows significant correlations between BMI levels (x axis) and average SPM-T values of glucose metabolism in a series of *a priori* selected ROIs (y axis), in the female cohort. Positive SPM-T values indicate higher-than-average brain glucose metabolism in each ROI, as obtained through comparison with a reference control sample [see text]. Higher BMI levels are associated with increased glucose metabolism. Only ROIs where correlation is significant after Bonferroni correction are shown. Gray shaded areas represent confidence intervals for the regression line slope.

Results of the ROI-based multivariate ANCOVA are reported in Table 2 and Figure 2. In the male group, BMI levels did not significantly predict brain metabolism in any of the *a priori* selected brain regions. In contrast, in the female group, BMI significantly predicted brain metabolism in the right gyrus rectus and right superior orbitofrontal gyrus (p<0.05, surviving Bonferroni-correction for multiple comparisons). Additional results, although not surviving Bonferroni-correction, were present in further regions related to a) cognitive control/decision making processes (i.e. left and right medial orbitofrontal cortex; right dorsolateral prefrontal cortex), b) salience attribution (i.e. left and right superior orbitofrontal gyrus; right middle orbitofrontal gyrus; right anterior insula), c) gustatory integration (i.e. right anterior and posterior insula)(Table 2). In the above-mentioned brain regions, whereas female subjects with normal BMI levels presented with normal regional metabolism values (T≈0), female subjects with higher BMI levels showed increased regional metabolism (T>0) (Figure 2).

**Table 2:** Results of the multivariate ANCOVA on regional metabolism.

**ROI**	**BMI - Males**			**BMI -Females**			**Gender x BMI Interaction**	
	***F***	***P-value***	***R***	***F***	***P-value***	***R***	***F***	***P-value***
*L Dorsolateral Prefrontal Cortex*	1.59	0.208	0.11	2.59	0.111	0.17	-	-
*R Dorsolateral Prefrontal Cortex*	2.86	0.092	0.14	4.22	0.041*	0.19	-	-
*L Medial Orbitofrontal Cortex*	0.38	0.540	0.04	3.96	0.048*	0.19	-	-
*R Medial Orbitofrontal Cortex*	1.79	0.182	0.11	4.61	0.033*	0.20	-	-
*L Gyrus Rectus*	0.77	0.381	0.07	2.60	0.108	0.17	-	-
*R Gyrus Rectus*	0.55	0.461	0.05	15.79	<0.001**	0.41	3.75	0.054°
*L Anterior Cingulate Cortex*	2.03	0.156	0.11	2.04	0.155	0.13	-	-
*R Anterior Cingulate Cortex*	2.27	0.133	0.11	2.11	0.148	0.12	-	-
*L Superior Orbitofrontal Gyrus*	0.80	0.373	0.07	8.00	0.005*	0.26	3.62	0.058°
*R Superior Orbitofrontal Gyrus*	0.43	0.513	0.04	11.83	0.001**	0.35	6.47	0.012*
*L Middle Orbitofrontal Gyrus*	0.18	0.670	0.03	3.58	0.06°	0.18	-	-
*R Middle Orbitofrontal Gyrus*	0.61	0.434	0.05	7.11	0.008*	0.25	3.49	0.063°
*L Inferior Orbitofrontal Gyrus*	1.97	0.162	0.11	1.56	0.214	0.15	-	-
*R Inferior Orbitofrontal Gyrus*	2.94	0.088	0.13	2.34	0.128	0.17	-	-
*L Anterior Insula*	0.06	0.814	0.01	0.54	0.462	0.08	-	-
*R Anterior Insula*	0.42	0.520	0.06	6.50	0.012*	0.22	2.73	0.100
*L Posterior Insula*	0.03	0.864	0.00	0.70	0.403	0.13	-	-
*R Posterior Insula*	1.37	0.244	0.09	5.47	0.020*	0.30	2.32	0.130
*L Ventral Striatum*	0.02	0.894	-0.01	1.25	0.265	0.14	-	-
*R Ventral Striatum*	0.19	0.663	0.02	3.01	0.084	0.23	-	-
*L Amygdala*	0.44	0.506	0.05	1.85	0.175	0.17	-	-
*R Amygdala*	0.13	0.717	0.02	1.53	0.217	0.14	-	-

F and p-values denote results of the multivariate ANCOVA on regional metabolism.

R values denote partial correlations between BMI and mean regional glucose consumption, factoring out the effects of age, cognitive status (as measured by MMSE) and years of education

**Significant correlations at p<0.05, after correction for multiple comparisons (N=22 ROIs) using Bonferroni correction

*Significant correlations at p<0.05, uncorrected for multiple comparisons

°Trend towards significance

At consistence with the voxel-based analysis, post-hoc analysis using ANCOVA revealed a significant BMI x gender interaction on metabolism of the right superior orbitofrontal gyrus (F=6.47, p=0.012), suggesting that the effect of BMI on orbitofrontal metabolism is significantly stronger in females as compared to males (Table 2). We also found that age significantly predicted metabolism in the orbitofrontal cortex (left and right superior orbitofrontal gyrus, right middle orbitofrontal gyrus: F=6.09; 15.57; 8.66; p=0.009, 0.000, 0.004, respectively) and right gyrus rectus in the female cohort; still, no significant BMI x age interaction was found in these regions (p<0.05). This confirms that age and BMI are both significant and *independent* predictors of metabolic function in the orbitofrontal regions.

### Brain metabolic connectivity patterns in subjects with normal vs. high BMI

**Figure 3:**
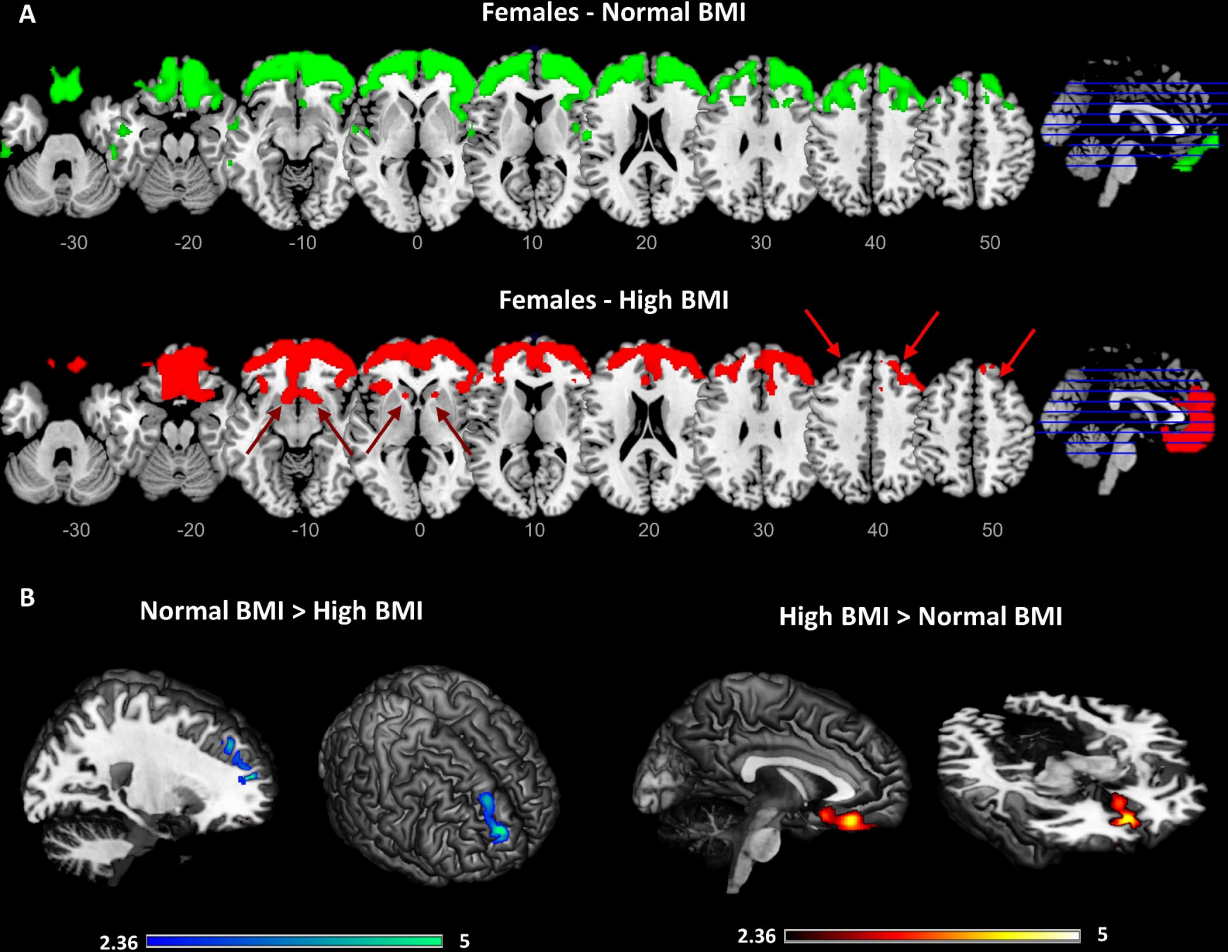
**Results of the data-driven metabolic connectivity analysis in females.** Figure shows results of the data-driven metabolic connectivity analysis, seeding from the BMI-related orbitofrontal cluster identified through whole-brain correlation analysis (see Figure 1 and text). The pattern of connectivity of the orbitofrontal cluster in females with normal BMI (upper panel) remarkably differs from the one observed in females with high BMI (lower panel) (**A**). In females with high BMI, loss of connectivity is evident between orbitofrontal cortex and high-order cortical regions, notably the dorsolateral prefrontal cortex (red arrows). Interconnections with reward-related brain circuits are also present (lacking in females with normal BMI), specifically involving the medial orbitofrontal cortex and nucleus accumbens (red arrows). Threshold for statistical significance was set at p<0.001 (uncorrected for multiple comparisons), minimum cluster extent k:100 voxels. Only clusters surviving SPM cluster-level FWE-correction (p<0.05) are shown Significant differences in connectivity strength between females with high BMI and females with normal BMI are also shown (p<0.01, uncorrected for multiple comparisons; p<0.05 at cluster-level; Ke: 100 voxels) (**B**). A high-resolution MRI anatomical template in MRIcron was used for rendering.

Results of the data-driven metabolic connectivity analysis are shown in Figure 3. Since a significant correlation between BMI and brain metabolism was found in females only, this metabolic connectivity analysis was restricted to this group. The metabolic networks seeding from the orbitofrontal cluster described above were remarkably different in females with normal vs. high BMI levels. Notably, in females with high BMI levels, the BMI-related cluster described above (i.e. right lateral orbitofrontal cortex, and rostro-polar portions of the right middle and superior frontal gyri) was significantly connected with the medial orbitofrontal cortex and gyrus rectus, bilaterally, and with the nucleus accumbens; this was not the case for females with normal BMI levels, where the BMI-related orbitofrontal cluster was significantly connected with large portions of the dorsolateral prefrontal cortex (more limited in females with high BMI levels) (p<0.001 at the voxel-level, p<0.05 FWE-corrected at cluster-level). Assessment of seed by BMI interactions confirmed that females with high BMI, compared to females with normal BMI, had significantly decreased connectivity in a cluster encompassing the right dorsolateral prefrontal cortex (cluster extent - Ke: 241 voxels) and significantly increased connectivity in a cluster encompassing the left medial orbitofrontal cortex (Ke: 334 voxels) (p<0.01 at the voxel-level, p<0.05 at cluster-level) (Figure 3).

Note that all the analyses described above included age, education and Mini-Mental State Examination (MMSE) as nuisance covariates. We did not correct for glycaemia levels, as no correlation was observed between BMI and fasting blood glucose in our case series (R=0.13, p=0.051; partial R=0.12, p=0.087), and no difference was observed also when comparing subjects with normal vs. high BMI, neither in the whole sample (T=0.836, p=0.404), nor in males (T=0.20, p=0.842) and females (T=0.884, p=0.379).

## DISCUSSION

Here, we report a female-specific effect of high BMI on brain metabolism and connectivity in the aged population. Specifically, high BMI was associated only in females with abnormally increased frontal metabolism and altered connectivity in executive, salience and reward systems, at consistence with the major neurocognitive theories of obesity [15]. On the contrary, in males, BMI was not correlated with focal brain metabolism changes. The present results are in line with previous data obtained in patients with Alzheimer’s disease, where high BMI was associated with altered connectivity in frontal and limbic brain networks in female patients, but not in males [16].

Our results indicate a major frontal dysfunction in overweight elderly female participants, as shown by a metabolic over-activation in brain regions specifically related to top-down control (dorsolateral prefrontal cortex and medial orbitofrontal cortex) and salience attribution and reward (lateral orbitofrontal cortex), that notably was reported in association with both overeating and obesity [17, 18] (Figures 1–2, Table 2). This effect was lateralized involving regions in the right hemisphere. Notably, only disruption of the activity of the right dorsolateral prefrontal cortex (and not the left), promotes disregard for choices with long-term adverse consequences [19]. Similarly, disruption of decision-making, and emotion processing was reported with damage to the right (but not the left) ventromedial prefrontal cortex [20]. This is also consistent with previous functional imaging studies reporting the right dorsolateral and ventromedial prefrontal cortex as involved in regulation of eating behavior [21].

In addition to the metabolic increases in the dorsolateral prefrontal cortex and orbitofrontal cortex, we also found increases in the frontopolar cortex and right insula (Figures 1–2, Tables 1–2). While the first is a key region for motivation, high-demand executive control, goal-directed behavior and reward monitoring [22], the latter is associated to food-related reward processes, all cognitive functions that have been reported as impaired in obesity [15]. Thus, dysfunction involves brain regions crucially involved in the top-down regulation of appetite (hedonic hunger), which is modulated by the dopaminergic reward system and is responsible for regulatory control over food intake, as opposed to the bottom-up regulation of appetite (metabolic hunger) underlying homeostatic metabolic regulation [23]. To this regard, a previous study on healthy adults (13 women and 10 men, age: 32.6±7.5 (22–48) years old, with BMI<30kg/m2) reported gender differences in brain metabolic activity related to top-down cognitive. inhibition of hunger during food presentation [11]. In adult men, but not in women, brain metabolism was modulated in limbic and paralimbic regions following active cognitive inhibition of hunger feelings [11]. The authors speculated that this might indicate a lack of inhibitory control of hunger and food-related behavior in women [11].

The second main finding concerns a large-scale reconfiguration of the executive and reward resting state networks in overweight/obese elderly females. Specifically, in females with high BMI, we found a loss of prefrontal-orbitofrontal connectivity, coupled with abnormal orbitofrontal interconnections with nucleus accumbens (Figure 3). This finding further contributes to previous evidence in obesity [24], suggesting that a long-lasting enhanced responsiveness and motivation for food stimuli may lead to a dysregulation of the prefrontal and orbitofrontal inhibitory control towards subcortical reward structures, in particular the nucleus accumbens [15]. All these interconnected regions are involved in the regulation of food intake, through the integration of both internal signals (i.e. homeostatic and cognitive state) and external factors (i.e. social environment) and have been found altered in obese/overweight individuals [25–27]. In particular, dysfunctions in the connectivity between these regions may reflect obesity-related defects in inhibitory control and attention processes involved in food intake behavior, and an increased motivation to internal signals, such as appetite or food-related reward [28]. Accordingly, previous studies have reported that women with obesity are characterized not only by increased resting-state activity in reward and salience brain regions [29] but also by enhanced functional connectivity between nucleus accumbens, anterior cingulate cortex and ventromedial prefrontal cortex [30]. In addition, in the fasting state, obese women as compared with lean female subjects, showed increased functional connectivity between the medial prefrontal cortex and other regions involved in cognitive control, motivation, and reward [31]. Considering this evidence, our results in elderly females suggest that high BMI levels affect metabolic connectivity between regions that are crucial for monitoring of internal and external stimuli, and that mediate emotional and affective control functions. Overall, our and previous findings suggest that in women with obesity, the food-related behavior may be led more by an imbalance in brain circuits related to reward-seeking and cognitive control, than by energy regulation and homeostasis maintenance. Notably, our results indicate that this dysfunctional obesity-related reconfiguration is also maintained in aging.

The gender difference demonstrated here in the association between BMI and brain metabolism brings several considerations for medical practice and health policy, considering that the neurophysiological mechanism(s) via which the reward circuits are different in males and female. It is conceivable that the difference relies on genetic and/or environmental factors. Genetic determinants for a gender difference in brain connectivity are likely to be mainly linked to sex chromosome variance. fMRI studies on girls with Turner syndrome and attention deficit hyperactivity disorder suggest a role of X-monosomy in affecting brain attention networks and cognitive function [32]. Furthermore, women with Complete Androgen Insensitivity Syndrome (CAIS), i.e. having a male karyotype (46, XY) but no functional androgen receptors, show lack of masculinization of the human brain [33]. Concerning phenotypical characteristics, administration of testosterone to middle-aged women diminished orbitofrontal cortex activity and its effective connectivity with the amygdala [33]. Vice versa, administration of estrogens to middle-aged postmenopausal women, is reported to increase the amygdala-prefrontal cortex connectivity [34] and enhance prefrontal cortex activity during cognitive control tasks [35]. Estrogen levels have also been shown to modulate response of brain regions that process emotion and reward signals, as measured by fMRI, in pre-menopausal women [36]. The development of central resistance to the effect of insulin, leptin as well as other hormones effective on energy metabolism and chronically altered in obesity may also explain the BMI-related differences in brain glucose metabolism [37, 38].

Theoretically, the association between obesity and frontal lobe dysfunction in the female elderly population may be partially also explained by the effect of aging on these regions. In late life, aging-related changes in frontal metabolic activity have been suggested to be at the basis of age-related impairment in executive functions [39]. This may lead to a major dysregulation in impulse control and eating behavior in elderly people, which may contribute to enhance the risk of overweight and obesity in late life. At consistence with this evidence, we indeed found that age was a significant predictor of metabolism in the orbitofrontal BMI-related cluster in the female cohort (F=6.446, p=0.012) (Figure 1C), as also confirmed by the ROI-based analysis. By performing an ANCOVA, to exclude a major effect of age *per se* on these results (namely that altered metabolism in females significant and *independent* predictors of metabolic function in orbitofrontal regions, and that their effects on metabolism combine in an additive fashion. Overall, these results confirm that BMI levels *per se* correlate with metabolism in brain regions that are also, and independently, affected by age.

Glycaemia level should also be taken into account as a possibly confounding factor on current results. It must be noted that no correlation was observed between BMI and fasting blood glucose (R=0.13, p=0.051; partial R=0.12, p=0.087) and that all individuals underwent [18F]FDG-PET examination with fasting blood glucose <160 mg/dL, at consistence with international guidelines [40]. Crucially, no difference in blood fasting glucose was observed in males vs. females (T=0.11, p=0.916), thus excluding glycaemia levels as underlying the gender-specific effects here reported. In addition, no difference was observed when directly comparing subjects with normal vs. high BMI, neither in the whole sample (T=0.84, p=0.404), nor in males (T=0.20, p=0.842) or females (T=0.88, p=0.379).

There are some limitations to our study. First, we acknowledge that, although, average BMI did not differ between males and females included in our case series, as shown by both parametric and non-parametric tests (T=1.09, p=0.279; U=5423, p=0.127), the prevalence of overweight subjects was higher in males than in females (χ^2^=8.87, p<0.05). Second, we have not applied partial volume correction to our metabolic imaging data, assuming also that these changes are still relatively limited in healthy elderly subjects; however, age was included as covariate in our analyses to exclude age-related brain changes from our results. Third, the cross-sectional nature of the study did not allow to define a direct causality between obesity and brain metabolic changes in aging. Last, BMI is the most widely established and used index for obesity, but it does not report any information on percent of body fat and its distribution, which may be additional characteristics to take into account by future studies.

In conclusion, for the first time we report BMI-related effects on brain metabolism in a healthy elderly population. Notably, in healthy elderly females high BMI correlates with glucose metabolism in brain areas involved in the executive network and reward system. Although our results cannot be generalizable to lifespan obesity-related effects, our findings seem to suggest gender-related differences in BMI effects on brain functioning in old age. Additional studies are needed to demonstrate a cause-effect relationship between high BMI and increased brain glucose metabolism, as well as the relevance of this finding to the management of elderly adults with obesity.

## MATERIALS AND METHODS

### Participants

Two hundred and twenty-two cognitively normal healthy elderly controls were retrospectively collected from the ADNI database. The ADNI was launched in 2003 as a public-private partnership, led by Principal Investigator Michael W. Weiner, MD. The primary goal of ADNI has been to test whether serial magnetic resonance imaging, PET, other biological markers, and clinical and neuropsychological assessment can be combined to measure the progression of mild cognitive impairment and early Alzheimer’s disease.

The group comprised 115 males and 107 females, with a mean age of 74.03±5.88 [61.2-85.9] years. Each subject underwent [18F]FDG-PET scan for the assessment of brain metabolism and a full neurological and neuropsychological evaluation. All subjects underwent a medical history revision and a psychiatric evaluation, and the presence of both diabetes and eating disorders were reported in medical notes. There was no record of eating disorders for the subjects included in our case series, while 14 out of 222 cases presented with diabetes type II. In all subjects, the glycaemia at the time of PET scan was less than 160 mg/dl, as recommended by the international guidelines [40]. Cognitive status, as evaluated by means of the MMSE, was reported normal in all subjects (mean±SD=29.03±1.23 [26-30]). BMI (calculated measuring height and body weight) data were collected concurrently with the [18F]FDG-PET scan. Average BMI was 27.00±4.02 [19.21-38.79] kg/m2, with 35.1% of subjects having a normal BMI, 43.7% being overweight and 21.2% being obese. There were no significant differences in mean (T=1.09, p=.279) and median BMI (U=5423, p=0.127) values between males and females. In females, 43% (N=46) were normal-weight, 33.6% (N=36) were overweight, 23.4% (N=25) were obese. Correspondingly, in males 27.8% (N=32), 53% (N=61), and 19.1% (N=22) were normal, overweight or obese, respectively. World Health Organization cut-offs points were used for subject classification into normal-weight (18.50≤BMI≤24.99), overweight (25≤BMI≤29.99) and obese (BMI≥30) categories (http://www.euro.who.int). Demographic and clinical characteristics are reported in Table 3. All subjects provided written informed consent; the protocols conformed to the Ethical standards of the declaration of Helsinki for protection of human subjects.

**Table 3:** Demographic and clinical characteristics (mean ± standard deviation; range) for the whole group and male and female groups, and significance of one-sample chi-square and independent samples t-tests for males vs. females comparisons.

	**Whole group**	**Males**	**Females**	**Test statistic**	**P-value**
**N**	222	115	107	0.29	0.59
**Age (years)**	74.03 ± 5.88 (61.2-85.9)	74.83 ± 6.19 (61.2-85.6)	73.17 ± 5.44 (62.0-85.9)	2.11	0.04^*^
**MMSE**	29.03 ±1.23 (26-30)	28.95 ± 1.31 (26-30)	29.11 ± 1.14 (26-30)	0.99	0.32
**Education(years)**	16.34 ± 2.74 (8-20)	17.10 ± 2.75 (8-20)	15.52 ± 2.50 (10-20)	4.45	<0.001^**^
**Blood fasting glucose (mg/dL)**	100.98 ± 15.71 (59-154)	100.63 ± 15.18 (66-152)	101.35 ± 16.32 (59-154)	0.34	0.74
**BMI (kg/m2)**	27.00 ± 4.02 (19.22-38.79)	27.28 ± 3.49 (19.22-37.35)	26.69 ± 4.52 (19.75-38.79)	1.09	0.28

Abbreviations: MMSE: Mini Mental State Examination; BMIL body mass index.

* = p<0.05, **= p<0.001 Significant difference at the two-sample t-test comparing males and females

### [18F]FDG-PET acquisition and pre-processing

[18F]FDG-PET acquisition procedure is described in the “ADNI PET technical procedures manual, version 9.5” (http://adni.loni.usc.edu/wp-content/uploads/2010/ 09/PET-Tech_Procedures_Manual_v9.5.pdf). First, a sequence of three 5-minute frames, starting at 45 minutes after radio-ligand injection, was combined into a single averaged image. As for pre-processing, each [18F]FDG-PET image was spatially normalized to a specific [18F]FDG-PET template in the MNI space [41]. Normalized images were written with an isotropic voxel size of 2 mm, and spatially smoothed with an isotropic 3D Gaussian kernel (FWHM: 8-8-8 mm). Intensity normalization was achieved by dividing each image by its global mean, in order to reduce inter-subject and inter-scanner variability. Image pre-processing was performed using SPM5 software (http://www.fil.ion. ucl.ac.uk/spm/software/spm5/), running in Matlab (MathWorks Inc., Sherborn, MA, USA).

Different analyses were performed with the following rationale: first, we used an exploratory whole-brain approach to test the correlation between BMI level and voxel-wise brain metabolism. Subsequently, we performed a hypothesis-driven ROI-based analysis, testing for the correlations between BMI levels and specific brain regions, selected on the basis of previous neuroimaging evidence in obesity [42, 43]. Finally, considering that high BMI levels might be associated not only to altered local metabolism, but also to long-distance connectivity dysfunctions, we tested for differences in metabolic connectivity in the BMI-related brain regions identified in the previous analysis.

### Association between brain metabolism and BMI

### Voxel-based analysis

First, we adopted an exploratory whole-brain approach, to test, without any *a priori* assumption, for gender-specific associations between BMI and voxel-wise brain glucose metabolism, as measured by [18F]FDG-PET. An ANCOVA model was implemented in SPM to assess the association between brain glucose metabolism and BMI, in females and males. Age, MMSE scores and years of education were entered as nuisance covariates. Statistical thresholds of p<0.001 at the voxel-level, p<0.05 FWE-corrected at cluster-level (minimum cluster extent Ke:100 voxels), were deemed as a reasonable trade-off between statistical robustness and sensitivity [44]. Post-hoc analyses were run to further characterize the results obtained in the first round of analysis, testing whether the correlation between BMI and average glucose metabolism in the BMI-related clusters (identified with the voxel-wise analysis described above) was modulated by gender and by age. This was done by implementing an ANCOVA model in SPSS, including a gender x BMI and age x BMI interaction as predictors of average metabolism in the BMI-related clusters.

For results interpretation and visualization purposes, [18F]FDG-PET metabolism values were then converted into T-score values, by performing a two-sample T-test between each single subject in our case series and a reference sample of healthy controls (see for example [45]). T-score values provide a measure of the degree of metabolic *alteration* at the single-subject level, as obtained from the head-to-head comparison of the subject with a large database (N=112) of elderly healthy controls (age= 64.68±9.35 (28-83) years; gender (M/F)= 59/53). Each [18F]FDG-PET image scan was tested for relative hyper- and hypo-metabolism by comparison with the reference group of 112 controls on a voxel- by-voxel basis using the general linear model, by means of the two-sample t-test design, in SPM5. Age was included as a covariate. A T-score of 1.65 (or -1.65) equals to the critical T value for hypermetabolism (or hypometabolism) at a liberal threshold of 0.05 (uncorrected for multiple comparison) with ν≥30. T-score values greater than 1.65 can be considered as hyper-metabolic, whereas T-score values under -1.65 can be considered as hypo-metabolic.

### Regions of interest (ROI)-based analysis

Second, we performed a hypothesis-driven ROI-based analysis, testing for gender-specific effects of BMI on brain glucose metabolism in a series of brain regions, *a priori* selected based on well-established neuroimaging evidence in obesity [15]. Specifically, we considered the following ROIs: the dorsolateral prefrontal cortex and medial orbitofrontal cortex, including the gyrus rectus (involved in control-decision making processes), the lateral orbitofrontal cortex, including the superior, middle and inferior orbitofrontal gyri (salience attribution), the anterior cingulate cortex (control-decision making processes/salience attribution), the insula, in its anterior and posterior portions (interoception and gustatory integration), ventral striatum (reward processing), and amygdala (emotional learning) (see Table 2). We used the Automated Anatomical Labelling (AAL) Atlas to derive the aforementioned ROIs [46], plus, in order to properly address specific sub-regions, the Sallet’s Dorsal Frontal Parcellation Atlas [47] for the dorsolateral prefrontal cortex, the Jülich histological atlas [48] for the anterior and posterior insula, and the boundaries provided by Tziortzi and colleagues (2011) [49] for the ventral striatum. A multivariate ANCOVA model was run, entering average brain glucose metabolism in the selected ROIs as dependent variables. Age, MMSE scores and years of education were entered as nuisance covariates. A post-hoc analysis was run, testing for gender x BMI and age x BMI interactions in the regions where a significant correlation between BMI and glucose metabolism was found. For results interpretation and visualization purposes, T-score values were also computed and used for visualizing significant results (see above).

### Brain metabolic connectivity analysis

In order to evaluate whether high BMI is coupled to defects in brain networks, we investigated the association between BMI levels (normal vs. high) and brain metabolic connectivity, by means of seed-based interregional correlation analysis [51]. This method, specifically validated for [18F]FDG-PET data [50], builds on the core principle that brain regions whose metabolism is correlated at rest are functionally interconnected [52]. Specifically, it allows to investigate patterns of connectivity at the group-level, by testing for the correlation between [18F]FDG-PET regional mean uptake of *a priori* selected seeds and voxel-wise [18F]FDG-PET glucose metabolism in the whole-brain. We consider, as seeds, the significant BMI-related clusters resulting from the voxel-based correlation analysis reported above, to evaluate the brain connectivity alterations stemming from core BMI-related regions. The averaged cluster uptake was set as variable of interest in an ANCOVA model in SPM, entering BMI level (normal vs. high) as fixed factor, and age, MMSE scores and years of education as nuisance covariates. Statistical threshold was set at p<0.001 at the voxel-level, and p<0.05 FWE-corrected at cluster-level (minimum cluster extent k:100 voxels). In order to investigate differences in connectivity strength between normal vs. high BMI groups, a post-hoc analysis was run to test for significant seed by BMI interactions within the BMI-related network estimated above. A more liberal threshold of p<0.01 at the voxel-level, and p<0.05 at cluster-level (minimum cluster extent k:100 voxels) was selected for this analysis.
